# Supplementary material for: The Wnt Co-Receptor PTK7/Otk and Its Homolog Otk-2 in Neurogenesis and Patterning
Source: Cells. 2024 Feb 20;13(5):365. doi: 10.3390/cells13050365 (PMC10930971; doi:10.3390/cells13050365)
Supplement: Supplementary file 1 [file cells-13-00365-s001.zip › cells-2756098-supplementary.pptx]

## Slide 1
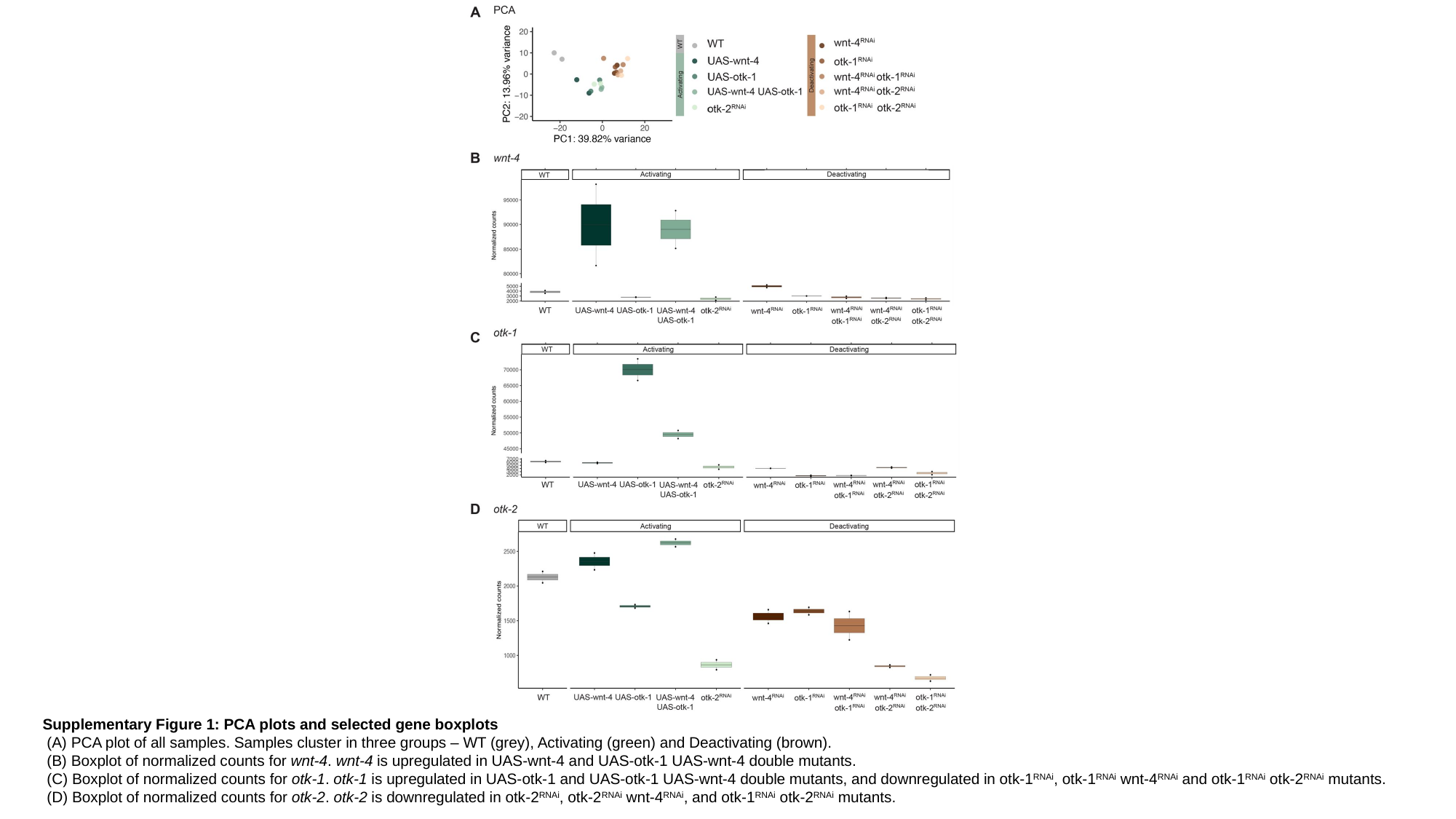

Supplementary Figure 1: PCA plots and selected gene boxplots
 (A) PCA plot of all samples. Samples cluster in three groups – WT (grey), Activating (green) and Deactivating (brown).
 (B) Boxplot of normalized counts for wnt-4. wnt-4 is upregulated in UAS-wnt-4 and UAS-otk-1 UAS-wnt-4 double mutants.
 (C) Boxplot of normalized counts for otk-1. otk-1 is upregulated in UAS-otk-1 and UAS-otk-1 UAS-wnt-4 double mutants, and downregulated in otk-1RNAi, otk-1RNAi wnt-4RNAi and otk-1RNAi otk-2RNAi mutants.
 (D) Boxplot of normalized counts for otk-2. otk-2 is downregulated in otk-2RNAi, otk-2RNAi wnt-4RNAi, and otk-1RNAi otk-2RNAi mutants.

## Slide 2
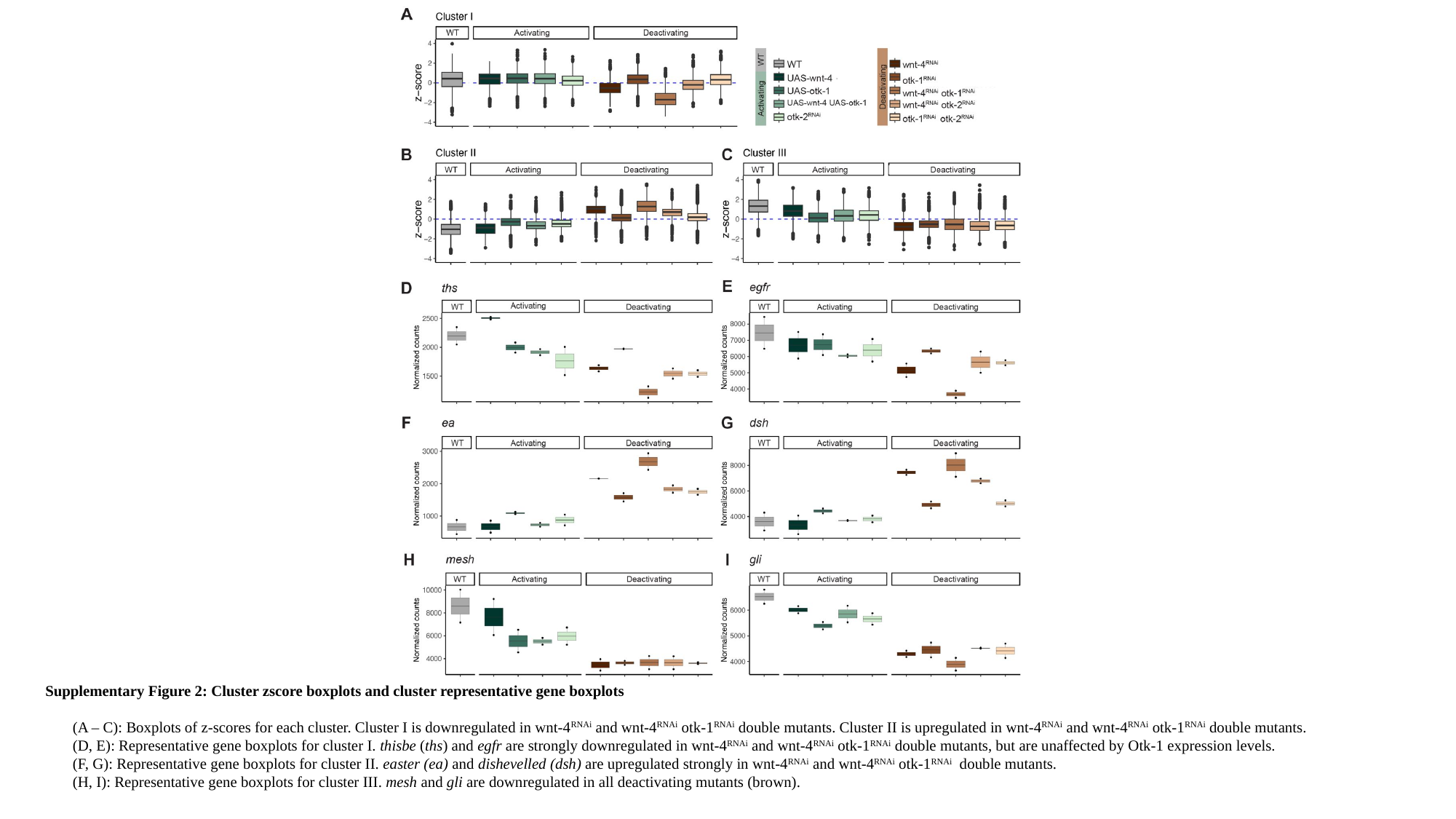

Supplementary Figure 2: Cluster zscore boxplots and cluster representative gene boxplots
(A – C): Boxplots of z-scores for each cluster. Cluster I is downregulated in wnt-4RNAi and wnt-4RNAi otk-1RNAi double mutants. Cluster II is upregulated in wnt-4RNAi and wnt-4RNAi otk-1RNAi double mutants.
(D, E): Representative gene boxplots for cluster I. thisbe (ths) and egfr are strongly downregulated in wnt-4RNAi and wnt-4RNAi otk-1RNAi double mutants, but are unaffected by Otk-1 expression levels.
(F, G): Representative gene boxplots for cluster II. easter (ea) and dishevelled (dsh) are upregulated strongly in wnt-4RNAi and wnt-4RNAi otk-1RNAi double mutants.
(H, I): Representative gene boxplots for cluster III. mesh and gli are downregulated in all deactivating mutants (brown).

## Slide 3
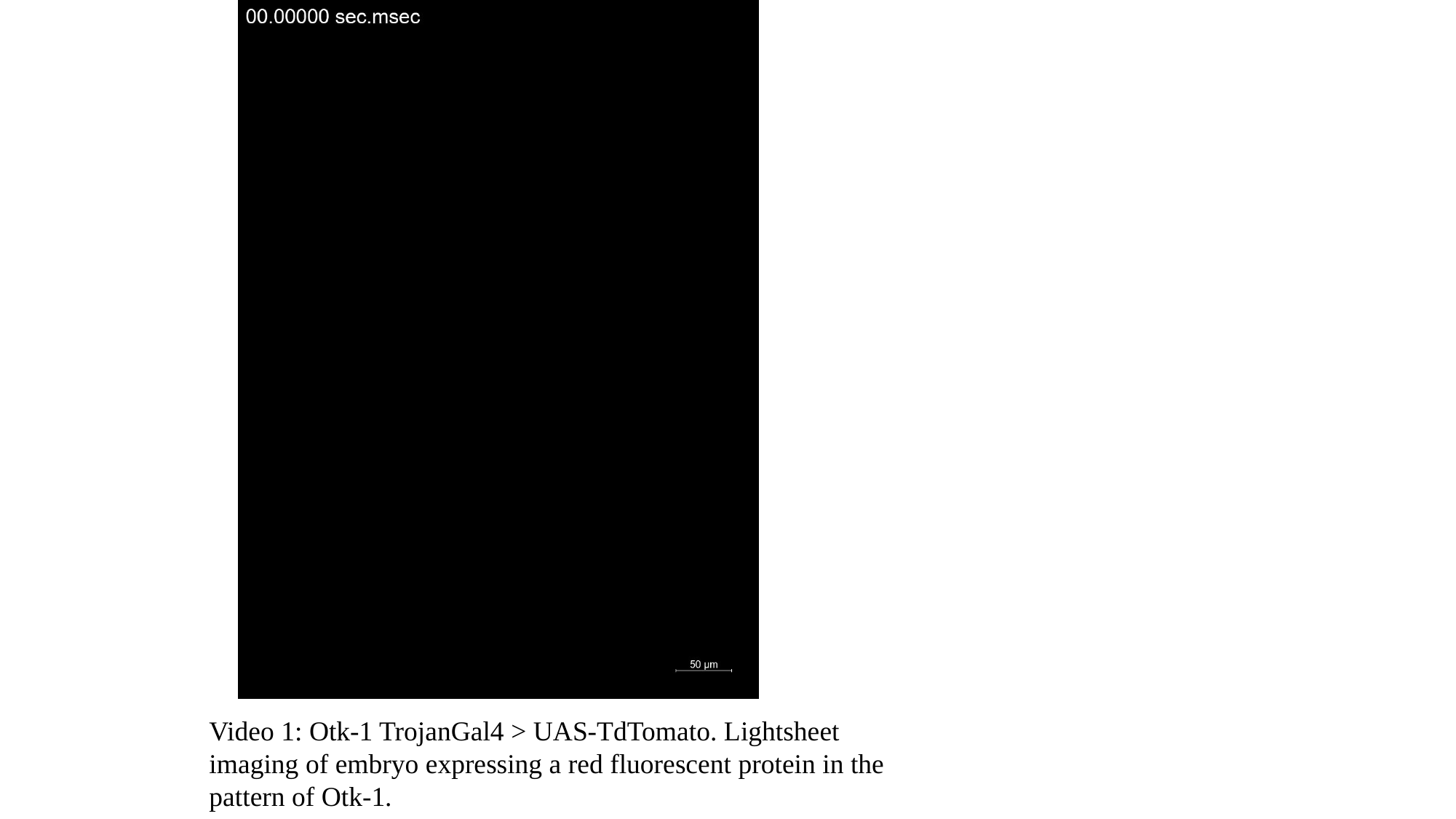

Video 1: Otk-1 TrojanGal4 > UAS-TdTomato. Lightsheet imaging of embryo expressing a red fluorescent protein in the pattern of Otk-1.

## Slide 4
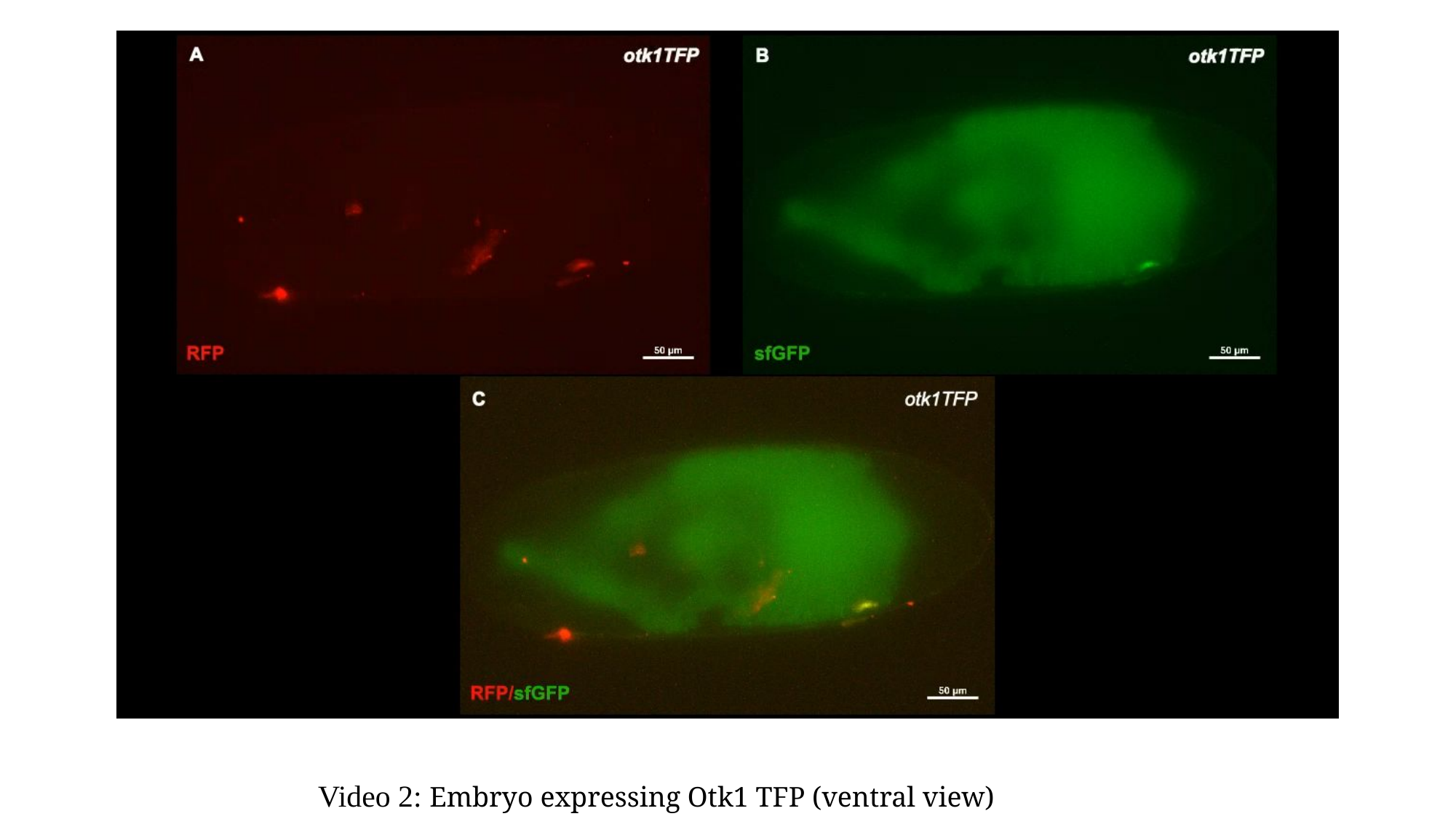

Video 2: Embryo expressing Otk1 TFP (ventral view)

## Slide 5
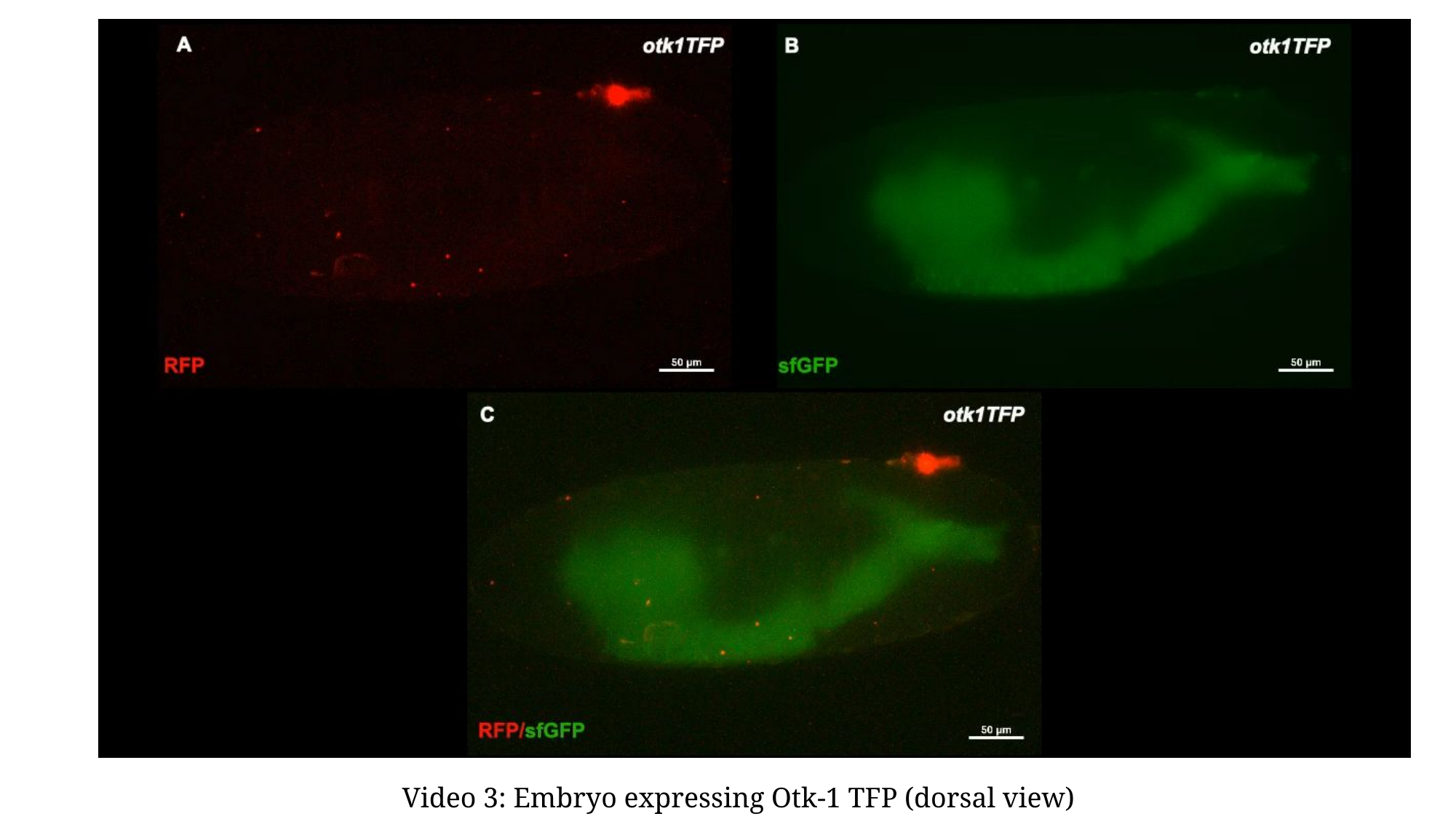

Video 3: Embryo expressing Otk-1 TFP (dorsal view)

## Slide 6
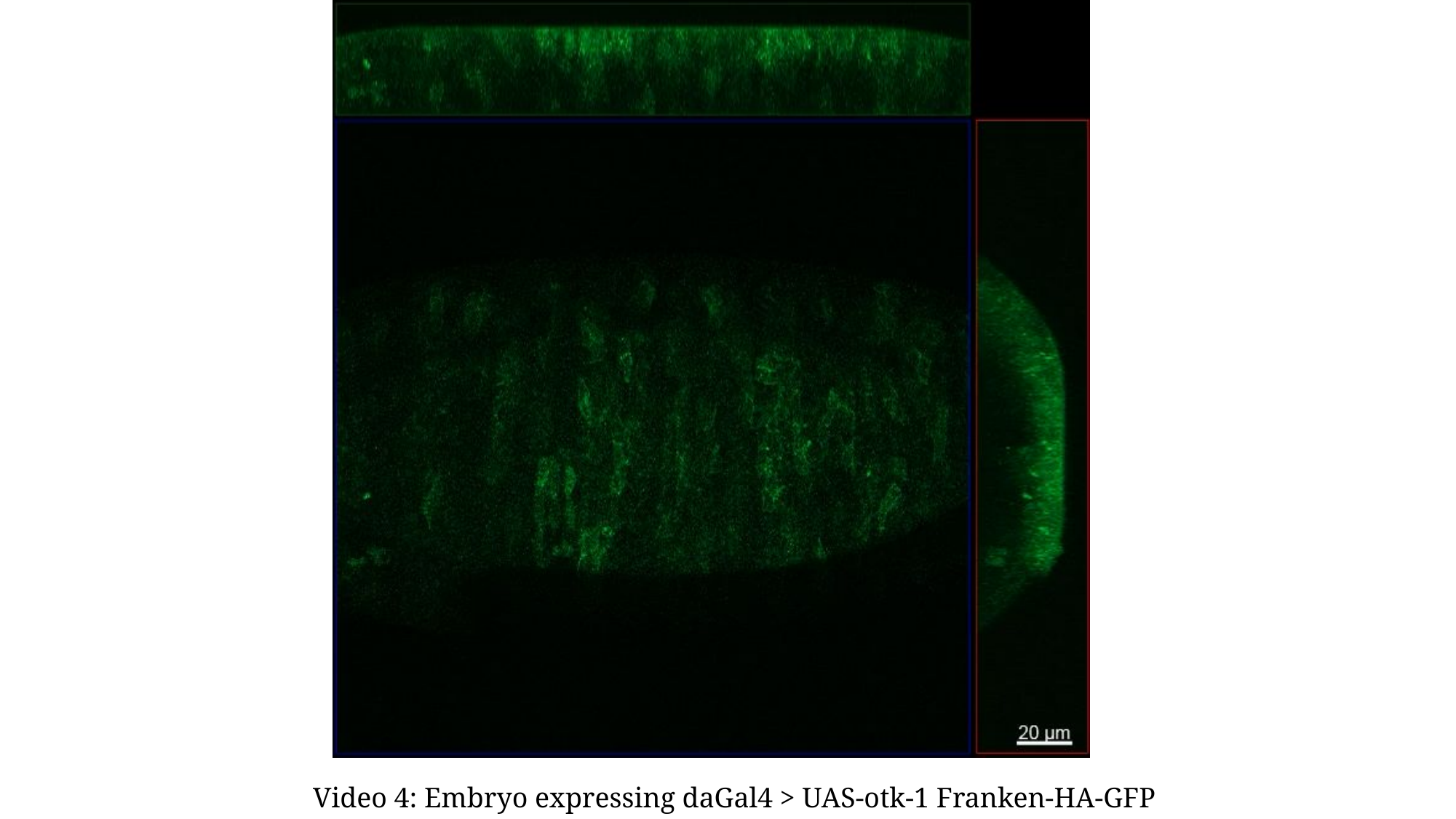

Video 4: Embryo expressing daGal4 > UAS-otk-1 Franken-HA-GFP

## Slide 7
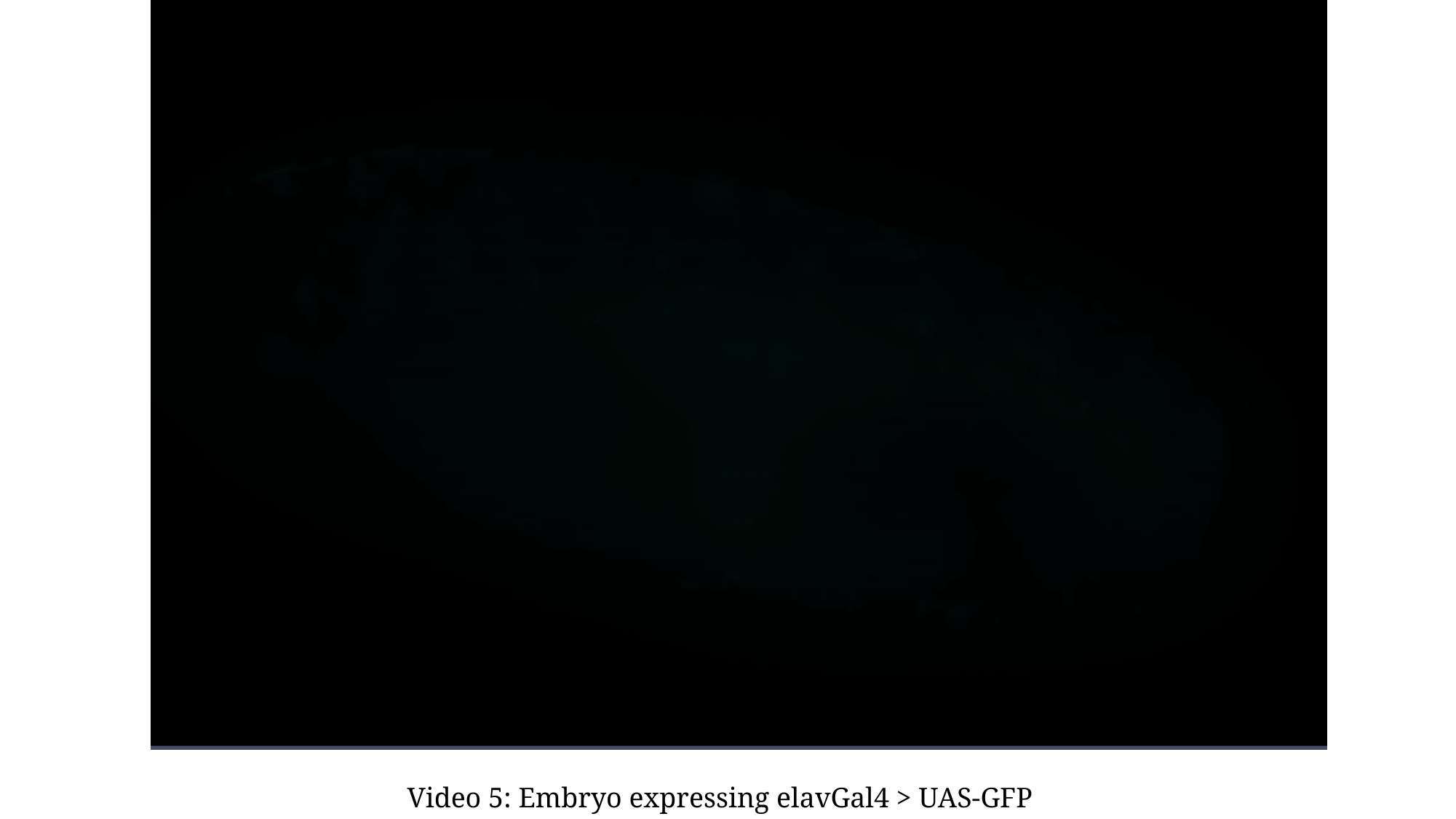

Video 5: Embryo expressing elavGal4 > UAS-GFP

## Slide 8
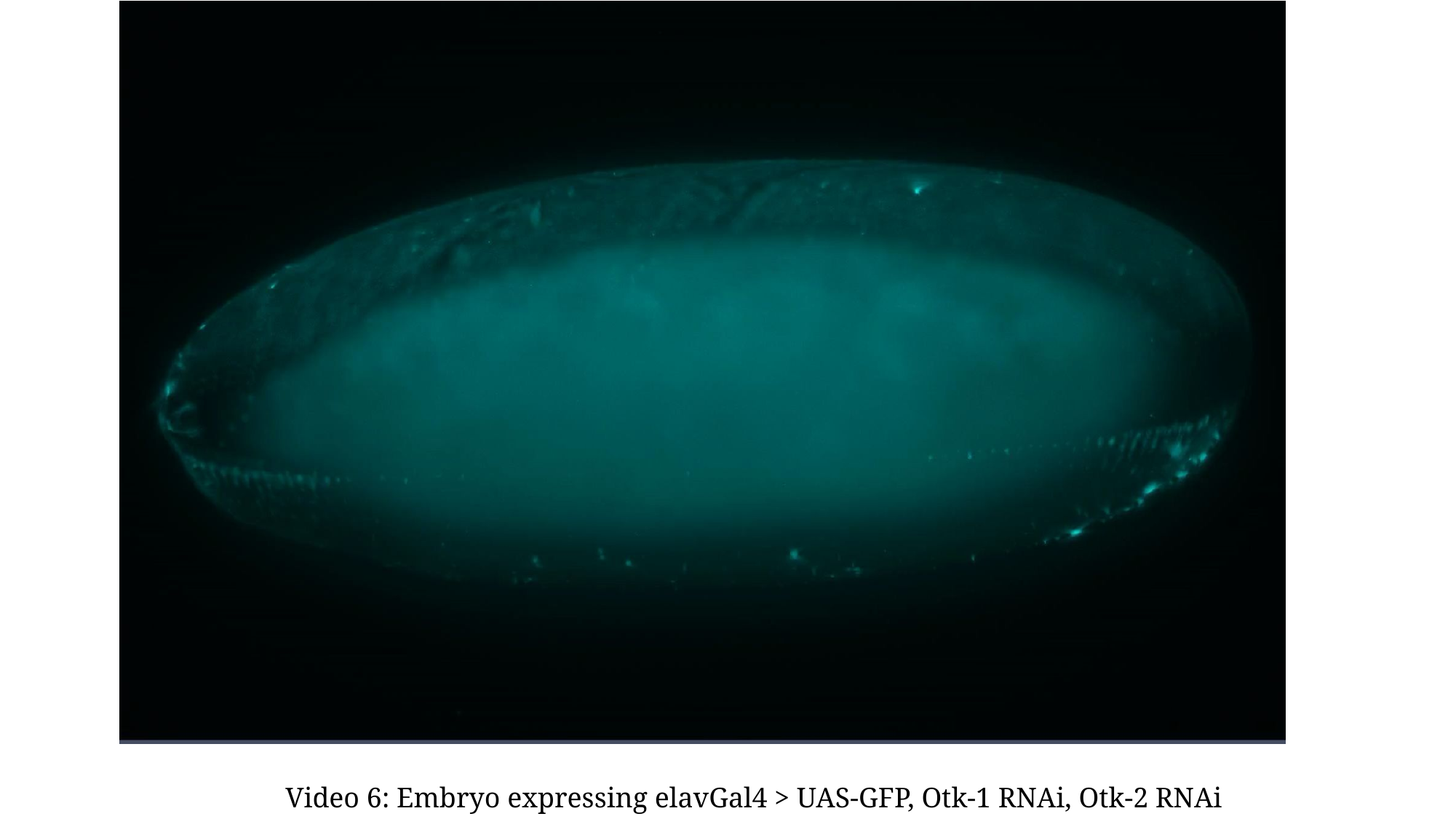

Video 6: Embryo expressing elavGal4 > UAS-GFP, Otk-1 RNAi, Otk-2 RNAi
